# Supplementary material for: The aging epigenome: integrative analyses reveal intersection with Alzheimer’s disease
Source: GeroScience. 2026 Apr 14;48(3):3185–203. doi: 10.1007/s11357-026-02195-x (PMC13356189; doi:10.1007/s11357-026-02195-x)
Supplement: Supplementary file 1 — (PDF 254 KB) [file 11357_2026_2195_MOESM1_ESM.pdf]

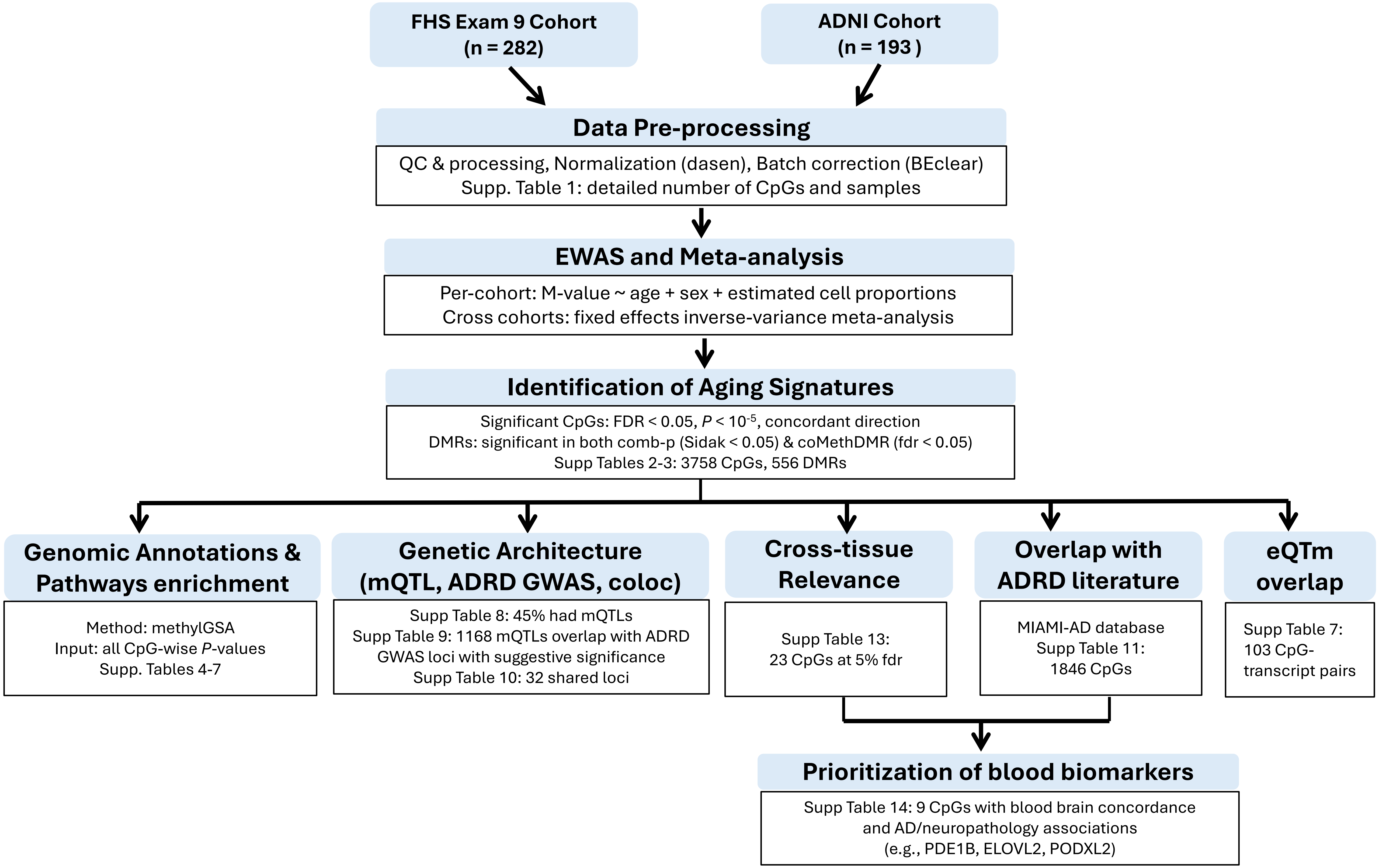

Supplementary Figure 1 Study workflow for identifying late-life blood DNA methylation (DNAm) aging signatures and intersection with Alzheimer's disease (AD).Blood DNAm (Illumina EPIC) from dementia-free participants aged ≥65 years in two cohorts (FHS Exam 9, n=282; ADNI, n=193) were processed using per-cohort quality control (QC), normalization, and batch correction. Epigenome-wide association studies (EWAS) were performed within each cohort using linear models (CpG M-values ~ age + sex + cell proportions) with correction for genomic inflation (bacon). Cohort-specific results were combined by fixed-effects inverse-variance meta-analysis to identify significant age-associated CpGs (FDR<0.05, $P<1\times10^{-5}$, concordant direction) and differentially methylated regions (DMRs) defined by intersection of comb-p and coMethDMR. Significant loci were interpreted via functional annotation and pathway enrichment, genetic integration (mQTLs, ADRD GWAS overlap, colocalization), cross-tissue brain–blood DNAm correlation, and overlap with independent AD DNAm studies, yielding a prioritized set of candidate blood-based biomarkers.

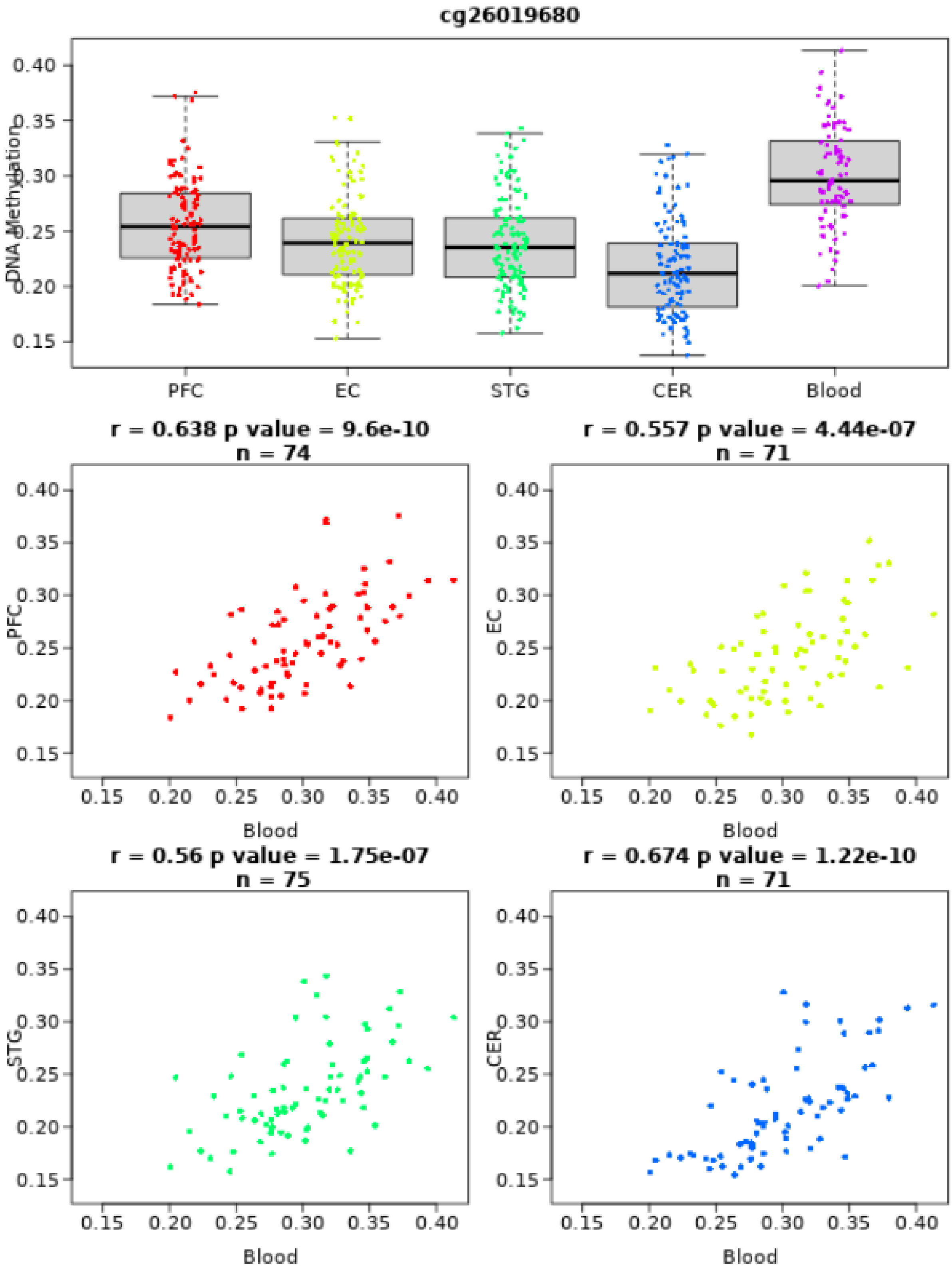

Supplementary Figure 2 Brain-blood correlations for DNA methylation levels at cg26019680 located in promoter region of the PODXL2 gene. These figures were obtained using the Blood Brain DNA Methylation Comparison Tool (https://epigenetics.essex.ac.uk/bloodbrain/?probenameg=cg26019680). **Abbreviations** PFC: Prefrontal Cortex, EC: Entorhinal Cortex, STG: Superior Temporal Gyrus, CER: Cerebellum
